# A qualitatively validated mathematical-computational model of the immune response to the yellow fever vaccine

**DOI:** 10.1186/s12865-018-0252-1

**Published:** 2018-05-25

**Authors:** Carla R. B. Bonin, Guilherme C. Fernandes, Rodrigo W. dos Santos, Marcelo Lobosco

**Affiliations:** 1 Graduate Program in Computational Modeling, Federal University of Juiz de Fora, Juiz de Fora, 36036-900 Brazil; 2 Presidente Antônio Carlos University - Medical School, Juiz de Fora, 36047-362 Brazil

**Keywords:** Computational vaccinology, Yellow fever, Mathematical modeling, Computational modeling, Immune system, Ordinary differential equations

## Abstract

**Background:**

Although a safe and effective yellow fever vaccine was developed more than 80 years ago, several issues regarding its use remain unclear. For example, what is the minimum dose that can provide immunity against the disease? A useful tool that can help researchers answer this and other related questions is a computational simulator that implements a mathematical model describing the human immune response to vaccination against yellow fever.

**Methods:**

This work uses a system of ten ordinary differential equations to represent a few important populations in the response process generated by the body after vaccination. The main populations include viruses, APCs, CD8+ T cells, short-lived and long-lived plasma cells, B cells and antibodies.

**Results:**

In order to qualitatively validate our model, four experiments were carried out, and their computational results were compared to experimental data obtained from the literature. The four experiments were: a) simulation of a scenario in which an individual was vaccinated against yellow fever for the first time; b) simulation of a booster dose ten years after the first dose; c) simulation of the immune response to the yellow fever vaccine in individuals with different levels of naïve CD8+ T cells; and d) simulation of the immune response to distinct doses of the yellow fever vaccine.

**Conclusions:**

This work shows that the simulator was able to qualitatively reproduce some of the experimental results reported in the literature, such as the amount of antibodies and viremia throughout time, as well as to reproduce other behaviors of the immune response reported in the literature, such as those that occur after a booster dose of the vaccine.

## Background

Mathematical and computational modeling is constantly evolving tool, which can be applied to many distinct research areas, such as Biology, Physics, Chemistry, Engineering, Biomechanics, Climate Modeling, tsunami and earthquake prediction, among others [1–23]. With this type of tool, the phenomenon under study is represented by mathematical equations which can be solved using computational simulators. The use of such mathematical-computational models can help reduce costs, time, risks and volunteers involved in the research. However, to achieve these objectives, the models must be very reliable. Since models are always an abstraction of reality, using simplifications to deal with complexities, many factors that can contribute to the real phenomenon may be ignored.

Mathematical models have been used for many years to represent various aspects of the immune system and related pathologies, but their application to describe the effects of vaccines has been rather limited [24]. The term computational vaccinology has been used to refer to computer-aided vaccine design [25–28], and its objective is to use different modeling techniques to aid the development and improvement of vaccines at different stages of their design processes.

In 1796 Edward Jenner introduced vaccination against smallpox, which was a major health problem at the time. Jenner observed that milkmaids were protected from smallpox after having suffered from cowpox, and concluded that cowpox could be used as a deliberate mechanism of protection against smallpox [29]. Jenner inoculated an 8-year-old boy, James Phipps, with cowpox. Subsequently, Jenner inoculated the boy again, this time with smallpox, and he did not contract the disease [29]. Jenner concluded that protection was complete. This is the key of vaccination: expose the body to antigens from pathogens, in order to stimulate the production of antibodies and defense cells against a specific disease.

Much of the work on computational vaccinology is related to the process of creating a new vaccine, such as in the selection of the best strains for use. In a previous paper [30] we proposed a new use for computational vaccinology, i.e. in the clinical development stage. With the use of mathematical and computational models, it is possible to experiment, *in silico*, different scenarios related to vaccination, to address important questions that remain unanswered.

The Yellow Fever (YF) vaccine, available since 1937 [31], is made from live attenuated virus still capable of triggering an immune response and inducing the production of antibodies and memory cells. Live-virus vaccines induce an immune response similar to that obtained with exposure to wild virus, but the risk of presenting characteristic symptoms of the disease and its complications, or death, due to vaccination, is extremely small. The YF vaccine is considered an effective and safe vaccine, with high documented seroconversion rates and low rates of adverse events. It has been effectively used to control a non-eradicable disease and is one of the vaccines that can benefit from the new use of computational immunology. The reason for this is manifold.

Although recognized as an effective and safe vaccine, some questions remain unanswered or poorly understood, and could be reassessed using new technologies and tools. As the vaccine was developed decades ago, some steps of its developmental processes were established empirically. A good example is the optimal dose required for immunization. What is the vaccine dose with the best immunogenicity/reatogenicity ratio? There are clinical studies designed to evaluate this [32], but these studies require time and resources, and there are methodological restrains to test several different doses. With the use of mathematical and computational modeling techniques, it is possible to evaluate a larger spectrum of doses in a much shorter time, using far less resources.

Another controversial issue is the need for a booster dose. Using mathematical and computational modeling, it is possible to simulate, for an individual, what his/her antigen levels will be years in the future, in a few minutes, to assess the duration of immunity and the need for booster dose administration, taking into account differences among individuals and doses,to help in the design of prospective cohort studies.

Despite being considered a safe vaccine, there are rare serious adverse events that need to be reassessed, such as viscerotropic and neurotropic events. There are also questions regarding the safety for vaccinating specific populations such as the elderly, people living with Human Immunodeficiency Virus (HIV)/AIDS and other immunocompromised populations. Because the YF vaccine is a live-attenuated virus vaccine, there is a small but not insignificant risk of occasional higher viral replication related either to vaccine virus attenuation aspects or an inability of the immune system to control the vaccine virus replication.

Recently, YF outbreaks were recorded in Angola and the Democratic Republic of Congo (DRC), with the latest outbreak still underway in Brazil, starting in December 2016. From December 2016 to February 22, 2017, 1,345 suspected cases were recorded, of which 295 have been confirmed, and 215 deaths reported to the Brazilian Ministry of Health [33].

YF is not an eradicable disease because of its sylvatic cycle. Reported cases in the Brazilian outbreaks were classified as sylvatic YF, but the risk of urban YF reintroduction is imminent due to high levels of *Aedes aegypti* infestation in Brazilian cities where vaccination coverage is not routinely recommended. World stocks and YF vaccine production capacity are a logistic concerns which could impact the control of disease transmission, particularly in large outbreaks. In Kinshasa, capital of the DRC, fractional doses of the YF vaccine were administered for outbreak control [34].

Concerned about the risk of a global epidemic, the WHO launched in April 2017 a strategy called Eliminate Yellow fever Epidemics (EYE), which aims to eliminate YF epidemics in the world by 2026 [34]. Through early detection and rapid and appropriate response, it is possible to minimize suffering, damage and propagation [34]. This strategy has three goals: protect populations at risk, prevent the international spread of YF and contain outbreaks quickly. To achieve these goals, the strategy suggests actions on different fronts, including research and development of better tools and practices. Assessing data about optimal vaccine dose and duration of immunity could help the design of new vaccination strategies for disease control.

This work, therefore, presents a first step towards an ideal scenario to simulate distinct situations related to the use of the YF vaccine: a qualitatively validated mathematical-computational model of the immune response to the YF vaccine. The model considers the major populations of Human Immune System (HIS) cells and molecules important in the process of immunity acquisition, such as Antigen Presenting Cells (APCs), B and T lymphocytes, and antibodies, which are considered the main marker of immunity. The model was then evaluated using distinct scenarios, and was successful in qualitatively reproducing experimental results reported in the literature.

This work is organized as follows. First, Section Related works presents related works done in this fields. Section Methods presents the mathematical and computational models used to reproduce the immune system response to the YF vaccine. The results are then presented in Section Results and discussed in Section Discussion, and finally Section Conclusion presents our conclusions.

## Related works

The use of mathematical and computational models to help vaccine development is not new. In fact, several works use computational tools to aid vaccine design. For example, epitope-mapping algorithms have been used for vaccine design since the 1980s [35]. Since then, new computational tools have been used for selection of vaccine targets [36–44]. Most of the works focuses on using mathematical and computational tools to predict epitopes [45] or to develop virtual screening approaches (i.e, the identification of relevant antigens) [46–49]. This traditional use of computational vaccinology is related to pre-clinical development. This work focuses on the development of mathematical and computational models that can be used in the clinical development stage, i.e., when the vaccine is first tested in humans. We argue that it is possible to carry out some experiments *in silico*, reducing the search space for experiments in vivo or in vitro, and it is possible to eliminate, reinforce or weaken hypotheses and to propose new studies, thus saving time and resources.

Several computational modeling techniques applied to vaccination are analyzed and discussed by Pappalardo et al. [24]. The authors describe what mathematical and computational modeling are and how they can aid research in vaccination. Modeling is defined as human activity involving the representation, manipulation, and communication of everyday real world objects. In their review, two main types of modeling are considered: Agent-Based Models (ABM) and mathematical models. Mathematical models are mainly based on differential equations, whether ordinary or partial, with delayed and/or stochastic equations. In this work, we try to qualitatively validate a simplified mathematical-computational model of the immune response to the YF vaccine presented in a previous work [50], which is based on a live, attenuated viral strain. The model uses Ordinary Differential Equations (ODEs) to model the main cells and molecules related to adaptive immune response.

Another work uses an ODE-based approach to model the human immune response to vaccination against both YF and smallpox [51] using distinct data and equations sets, one for each disease. The aim of the authors was to primarily evaluate the dynamics of CD8+ T cells, while this work will evaluate the immune response as a whole. The model proposed here differs from that presented by Le et al. [51], since it considers important populations at each stage of the immune response to YF vaccination, from virus inoculation to APC antigen presentation and consequent activation of lymphocytes, generation of antibodies and memory cells.

## Methods

### Mathematical model

In this section, we present the model we proposed in a previous work [50], which will be qualitatively validated in this paper. The model consists of a system of 10 ODEs representing important populations in the response process generated by the body after vaccination. The main populations are viruses, APCs, CD8+ T cells, short-lived and long-lived plasma cells, B cells and antibodies. Only populations related to the YF vaccine are modeled. For example, only B and T cells whose receptors can recognize the YF virus are considered in the model.

Equation 1 represents the vaccine virus (*V*).

1 $$  \frac{d}{dt}V= \pi_{v} V - \frac{c_{v1} V}{c_{v2} + V} - k_{v1} V A - k_{v2} V T_{E}  $$

The virus can not proliferate by itself, it needs to infect a cell and use it as a factory for new viruses. This is implicitly considered in the term *π*_*v*_*V*, which represents the multiplication of the virus in the body, with a production rate of *π*_*v*_. The term $\frac {c_{v1} V}{c_{v2} + V} $ denotes a non-specific viral clearance by the innate immune system. This function is similar to the Hill family of equations [52].

$$ y = k_{1} \left (\frac{ x^{k_{2}}}{k_{3}^{k_{2}} + x^{k_{2}}}\right) $$

The above equation is a generalization of the hyperbolic saturation function. The parameter *k*_1_ scales the maximum value to which the function is asymptotic, *k*_2_ is a shape parameter and *k*_3_ is analogous to the half-saturation constant. If *k*_2_=1, the Michaelis-Menten function is produced [53].

The term *k*_*v*1_*VA* denotes specific viral clearance due to antibody signaling, where *k*_*v*1_ is the clearance rate. The term *k*_*v*2_*VT*_*E*_ denotes specific viral clearance due to the induction of apoptosis of cells infected by the YF virus, where *k*_*v*2_ is the clearance rate.

APCs are all cells that display antigens complexes on their surfaces, such as dendritic cells and macrophages. Two stages of APCs were considered: immature and mature. The first stage, immature APCs (*A*_*P*_), is described by Eq. 2.

2 $$  \frac{d}{dt}A_{P}= \alpha_{A_{P}} (A_{P0} - A_{P}) - \beta_{A_{P}} A_{P} \left(k_{A_{P1}} + tanh\left(V - k_{A_{P2}}\right)\right)  $$

The term $\alpha _{A_{P}} \left (A_{P0} - A_{P}\right)$ denotes the homeostasis of APCs, where $\alpha _{A_{P}}$ is the homeostasis rate. The term $\beta _{A_{P}} A_{P} \left (k_{A_{P1}} + tanh\left (V - k_{A_{P2}}\right)\right) $ denotes the conversion of immature APCs into mature ones. Therefore, the same term appears in Eq. 3 with positive sign. The constant $\beta _{A_{P}}$ represents the conversion rate and $\left (k_{A_{P1}} + tanh\left (V - k_{A_{P2}}\right)\right)$ is a sigmoidal saturation function in the form of a hyperbolic tangent.

Equation 3 represents the mature APCs (*A*_*PM*_).

3 $$ \frac{d}{dt}A_{PM}= \beta_{A_{P}} A_{P} \left(k_{A_{P1}} + tanh\left(V - k_{A_{P2}}\right)\right) - \delta_{A_{PM}}A_{PM}  $$

The first term, as explained, denotes the dynamics of APCs maturation. The second term, $\delta _{A_{PM}}A_{PM}$, denotes the natural decay of the mature APCs, where $\delta _{A_{PM}}$ is the decay rate.

Equation 4 represents the population of naïve CD8+ T cells (*T*_*N*_).

4 $$ \frac{d}{dt}T_{N}= \alpha_{T_{N}} \left(T_{N0} - T_{N}\right) - \pi_{T} A_{PM} T_{N}  $$

The term $\alpha _{T_{N}} (T_{N0} - T_{N})$ represents the homeostasis of CD8+ T cells, where $\alpha _{T_{N}}$ is the homeostasis rate. The term *π*_*T*_*A*_*PM*_*T*_*N*_ denotes the activation of naïve the CD8+ T cells, where *π*_*T*_ is the activation rate. Therefore, the same term appears in Eq. 5 with positive sign.

Equation 5 represents the effector CD8+ T cell population (*T*_*E*_).

5 $$ \frac{d}{dt}T_{E}= \pi_{T} A_{PM} T_{N} + k_{T_{E}} A_{PM} T_{E} - \delta_{T_{E}} T_{E}  $$

The term $k_{T_{E}} A_{PM} T_{E}$ represents the proliferation of effector CD8+ T cells. The term $\delta _{T_{E}} T_{E} $ represents the natural death of these cells, with $\delta _{T_{E}}$ representing its decay rate.

Equation 6 represents B cells (*B*), both naïve and effector ones. These populations were not considered separately in order to simplify the model.

6 $$\begin{array}{*{20}l} \frac{d}{dt}B= \alpha_{B} (B_{0} - B) + \pi_{B} A_{PM} B - \beta_{S} A_{PM} B \\ - \beta_{L} A_{PM} B - \beta_{B_{M}} A_{PM} B \end{array} $$

The term *α*_*B*_(*B*_0_−*B*) represents the B cells homeostasis, where *α*_*B*_ is the homeostasis rate. The term *π*_*B*_*A*_*PM*_ represents the proliferation of the active B cells. The terms *β*_*S*_*A*_*PM*_*B*, *β*_*L*_*A*_*PM*_*B* and $\beta _{B_{M}} A_{PM} B$ denote the portions of active B cells that differentiate into short-lived plasma cells, long-lived plasma cells and memory B cells, respectively. These terms will appear with positive sign in Eqs. (7), (8) and (9). The activation rates are respectively given by *β*_*S*_, *β*_*L*_ and $\beta _{B_{M}}$.

Equation 7 represents the short-lived plasma cells (*P*_*S*_).

7 $$  \frac{d}{dt}P_{S}= \beta_{S} A_{PM} B - \delta_{S} P_{S}  $$

The term *δ*_*S*_*P*_*S*_ denotes the natural decay of short-lived plasma cells, where *δ*_*S*_ is the decay rate.

Equation 8 represents the long-lived plasma cells (*P*_*L*_).

8 $$ \frac{d}{dt}P_{L}= \beta_{L} A_{PM} B - \delta_{L} P_{L} + \gamma_{M} B_{M}  $$

The term *δ*_*L*_*P*_*L*_ denotes the natural decay of long-lived plasma cells, with *δ*_*L*_ representing the decay rate. The term *γ*_*M*_*B*_*M*_ represents the production of these cells by memory B cells, where *γ*_*M*_ is the production rate.

Equation 9 corresponds to memory B cells (*B*_*M*_).

9 $$ \frac{d}{dt}B_{M}= \beta_{B_{M}} A_{PM} B + k_{B_{M1}} B_{M}\left(1 - \frac{B_{M}}{k_{B_{M2}}}\right) - \gamma_{M} B_{M}  $$

The term $k_{B_{M1}} B_{M}\left (1 - \frac {B_{M}}{k_{B_{M2}}}\right)$ represents the logistic growth of memory B cells, i.e., there is a limit to this growth. The constants $k_{B_{M1}}$ and $k_{B_{M2}}$ represent the growth rate and limits, respectively.

Equation 10 represents the antibodies. The terms *π*_*AS*_*P*_*S*_ and *π*_*AL*_*P*_*L*_ are the production of the antibodies by short-lived and long-lived plasma cells, respectively. The production rates are given by *π*_*AS*_ and *π*_*AL*_, respectively. The term *δ*_*A*_*A* denotes the natural decay of these cells, where *δ*_*A*_ is the decay rate.

10 $$  \frac{d}{dt}A= \pi_{AS} P_{S} + \pi_{AL}P_{L} - \delta_{A} A  $$

The model presented in this paper was based on an earlier study [54], which described a mathematical model to represent the human immune response to an infection by YF virus. Therefore, the first difference is that this paper focus on modelling the effects of the YF vaccine administered subcutaneously.

The previous work [54] modeled the immune response to the YF virus from infection of epithelial cells to secretion of antibodies, considering various populations of cells and molecules, in different stages and compartments. There were 19 ODEs divided into two compartments: one representing the tissue where the virus proliferates and the other the lymph nodes. In order to consider all the cells and molecules, the model became complex.

Another issue is related to its adjustment to reproduce some behaviors described in the literature: as the number of equations and parameters increases, so does the amount of data and information needed to adjust the model. The second difference between the two models is that the model reproduced here [50] reduces the number of equations from 19 to 10. The reduced model reproduced in this work considers only the main populations of cells and molecules involved in the response to the vaccine, and abstracts some details that are not crucial to represent the behavior of the immune response, such as the representation of distinct compartments. In addition, some populations were not considered because no experimental data are available to validate the simulations, such as CD4+ T cells. In the near future, more cells or molecules can be reintroduced in the model if their roles are important to explain or represent behaviors that the reduced model [50] could not represent. Table 1 summarizes the main differences between the model presented in previous work [54] and the one evaluated in this work.

**Table 1:** Main differences between the models

	Previous	Current
Number of equations	19	10
Number of parameters	54	27
Number of compartments	2	1
Number of populations considered	10	8

It is important to remember that a mathematical model is an abstraction of reality and therefore simplifications are always necessary. This is accentuated when the target of the model is the HIS response, a complex network that involves many tissues, organs and cells and that performs several processes. The level of abstraction depends on the purpose of the model. HIS can be seen at various levels, from the level of substances produced by cells, such as cytokines, to the level of cells and molecules, as in the case of the simplified model [50]. It also can reach the level of an entire population, as in the case of the epidemiological models. The use of a simplified model does not imply that it can reproduce only a limited number of scenarios. The point is that some of the aspects not directly included in the model may be indirectly present, as constants. As such, the choice of distinct values for some constants may represent distinct behaviors in the system.

### Computational model

For the resolution of the ODEs system, a code was implemented using Python programming language, which includes libraries for easily solving complex mathematical problems. The library chosen was SciPy [55]. This library has a package called “integrate”. One of the functions available in this package is called “odeint”, and it is used to numerically solve a system of ODEs. The choice of the numerical method to be used is made automatically by the function based on the characteristics of the equations. The function uses an adaptive scheme for both the integration step and the convergence order. The function can solve the ODEs system using either the Backward Differentiation Formula (BDF) or the Adams method [56]. BDF is used for stiff equations and the implicit Adams method is used otherwise.

The experiments were performed using Python version 2.7.10 using the Spyder Integrated Development Environment (IDE). The execution environment was composed by an Intel Core i5 1.6 GHz processor, with 8 GB of RAM. The system runs macOS Sierra version 10.12.5.

## Results

In order to qualitatively validate our model, four experiments were carried out. The first one simulates a scenario where an individual was vaccinated against YF for the first time. The standard dose of the vaccine was used in this scenario. The results of the simulation were then compared to experimental data obtained from the literature.

The second scenario assesses the immune response following the administration of a booster dose ten years after the first dose. The standard dose of the vaccine was also used in this scenario. Although there are no experimental data from the literature that could be used for comparison purposes, there is research reporting that the expected behavior is an individual to present a lower viremia, and to raise antibodies levels to levels higher than those obtained after the administration of the first dose [57].

The third scenario simulates the immune response to the YF vaccine in individuals with different levels of naïve CD8+ T cells prior to vaccination. This simulation aims to evaluate the importance of this population of cells in the control of viremia and in the production of antibodies.

The fourth scenario is based on an experimental study [32] in which distinct doses of the YF vaccine were tested. Compared to the standard dose, the experimental study reported that, to some extent, the reduction did not significantly affect the percentage of sero-conversion. In this scenario, computational experiments are executed several times, using distinct values for the vaccine doses. For comparison purposes, the computational experiments were carried out using the same values of the experimental study [32].

In addition to evaluating the response of the model when different doses are administered, we performed a sensitivity analysis of the parameters related to virus dynamics. The main results of this analysis are shown in subsection Sensitivity analysis.

All the initial values used for the variables as well as the model parameters are presented in Tables 2 and 3. The parameters were adjusted, except for *δ*_*A*_, whose value was extracted from the literature.

**Table 2:** Model variables and initial values

Variable	Description	Initial value
*V*	Vaccine virus	27,476
*A*_*P*_	Immature APCs	10^3^ ^*a*^
*A*_*PM*_	Mature APCs	0
*T*_*N*_	Naïve CD8+ T cells	10^3^ ^*a*^
*T*_*E*_	Effectors CD8+ T cells	0
*B*	B cells	10^3^ ^*a*^
*P*_*S*_	Short-lived plasma cells	0
*P*_*L*_	Long-lived plasma cells	0
*B*_*M*_	Memory B cells	0
*A*	Antibodies	0

Values marked with ^*a*^ were based on [68]

**Table 3:** Model parameters

Parameter	Equation	Description	Value
*π*_*V*_	1	Viral replication rate	4.0 (day^−1^)
*c*_*v*1_	1	Maximum viral clearance rate by the innate system	2 × 10^3^ (virus titer×day^−1^)
*c*_*v*2_	1	Half saturation constant	3 × 10^1^ (virus titer)
*k*_*v*1_	1	YFV neutralization rate per unit of neutralizing antibodies	4.875 × 10^−4^ (day^−1^×*A*^−1^)
*k*_*v*2_	1	YFV neutralization rate per unit of CD8 + T cells	1.5694 × 10^−3^$\left (\text {day}^{-1} \times T_{E}^{-1}\right)$
*α* *A*_*P*_	2	Homeostasis rate of immature APCs	2.5 × 10^−3^ (day^−1^)
$\beta _{A_{P}}$	2,3	APC maturation rate	3.0 × 10^−1^ (day^−1^)
$k_{A_{P1}}$	2,3	With $\beta _{A_{P}}$, defines the minimum production rate of *A*_*PM*_	1.0 (dimensionless)
$k_{A_{P2}}$	2,3	Defines the saturation point of *A*_*PM*_	2 × 10^2^ (virus titer)
$\delta _{A_{PM}}$	3	Death rate of mature APCs	5.38 × 10^−1^ (day^−1^)
$\alpha _{T_{N}}$	4	Homeostasis rate of CD8 + T cells	2.17 × 10^−4^ (day^−1^)
*π*_*T*_	4,5	Activation rate of *naïve* CD8 + T cells	1 × 10^−2^ (day^−1^)
$k_{T_{E}}$	5	Replication rate of effector CD8 + T cells	1 × 10^−5^ (day^−1^)
$\delta _{T_{E}}$	5	Death rate of effector CD8 + T cells	1 × 10^−1^ (day^−1^)
*α*_*B*_	6	Homeostasis rate of B cells	6.0 (day^−1^)
*π*_*B*_	6	Replication rate of active B cells	1.77 × 10^−3^ (day^−1^)
*β*_*S*_	6,7	Differentiation rate of active B cells in short-lived plasma cells	6.72 × 10^−1^ (day^−1^)
*β*_*L*_	6,8	Differentiation rate of active B cells in long-lived plasma cells	8.05 × 10^−3^ (day^−1^)
*β*_*BM*_	6,9	Differentiation rate of active B cells in memory B cells	1 × 10^−3^ (day^−1^)
*δ*_*S*_	7	Death rate of short-lived plasma cells	2.0 (day^−1^)
*δ*_*L*_	8	Death rate of long-lived plasma cells	2.22 × 10^−4^ (day^−1^)
*γ*_*M*_	8,9	Differentiation rate of memory B cells in long-lived plasma cells	1.95 × 10^−6^ (day^−1^)
$k_{B_{M1}}$	9	Proliferation rate of memory B cell	1 × 10^−5^ (day^−1^)
$k_{B_{M2}}$	9	Maximum growth constant	10.0 (*B*_*M*_)
*π*_*AS*_	10	Antibody secretion rate (short-lived plasma cell)	5 × 10^−1^ (day^−1^)
*π*_*AL*_	10	Antibody secretion rate (long-lived plasma cell)	1.7 × 10^−1^ (day^−1^)
*δ*_*A*_	10	Antibody death rate	4 × 10^−2^ (day^−1^)^*a*^

The value marked with ^*a*^ was extracted from [66, 67] *apud* [68]

In general, the literature reports two distinct sets of experimental data. The first one is viremia along time, i.e., the amount of virus present in the bloodstream. The second dataset reported in the literature is the antibody levels along time. Therefore, in order to validate the model, the values obtained by Eqs. 1 and (10) will be compared to experimental values found in the literature.

### First vaccination

This section presents the computational results of a simulation in which an individual was vaccinated against YF for the first time.

In this computational experiment, a value equal to 27,476 International Units (IU) was used as the standard amount of virus particles present in the vaccine. This value is set as the initial condition of the virus population represented in Eq. 1 by *V* (all initial conditions are presented in Table 2). In fact, in the case of the 17DD-YFV the amount of virus particles varies depending on the vaccine lot number, ranging from 2.3 to 12 times the minimum value required by the WHO [32]. The 17DD-YFV is the YF vaccine developed by Bio-Manguinhos/Fiocruz, one of the three producers prequalified by the WHO to supply vaccines to international agencies.

**Fig. 1:**
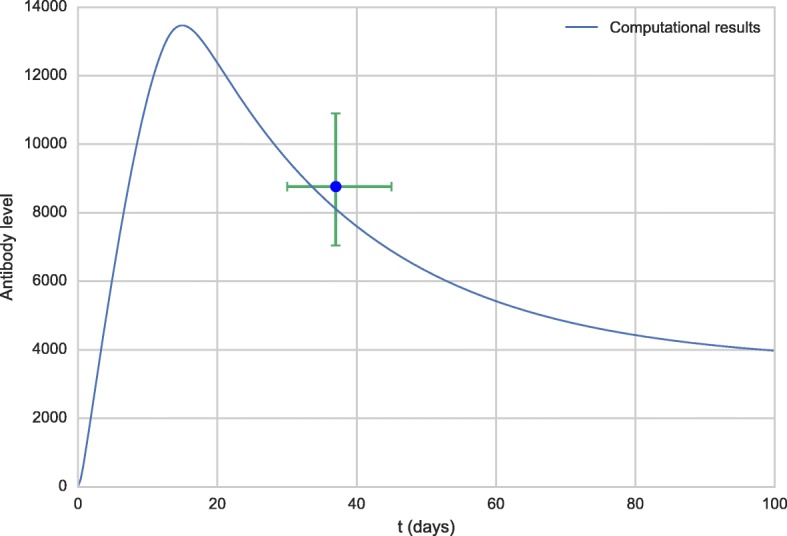
Antibody curve obtained by the model (line) and experimental data extracted from the literature [58] (dots)

**Fig. 2:**
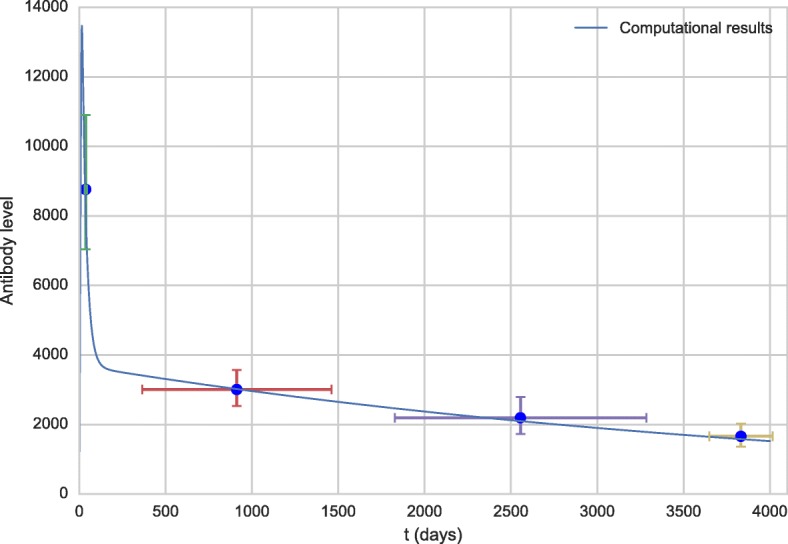
Antibody curve for the first 100 days obtained by the model (line) and experimental data extracted from the literature [58] (dots)

Figures 1 and 2 show the comparison of the antibody curve generated as a result at 100 and 4,000 simulation days, respectively, with the experimental results from the literature [58]. The result of the simulation is presented in separate figures in order to better observe the increase of the antibody level in the first days after vaccination.

The levels of antibodies obtained from the literature [58] are in Geometric Mean Titers (GMT) and refer to time intervals after vaccination. The time values used in the graph were obtained by averaging the times of each interval. For example, the first point was the 30-45 days post-vaccination interval, the value used was 37 days, the corresponding antibody level was 8,762.8 IU/mL.

**Fig. 3:**
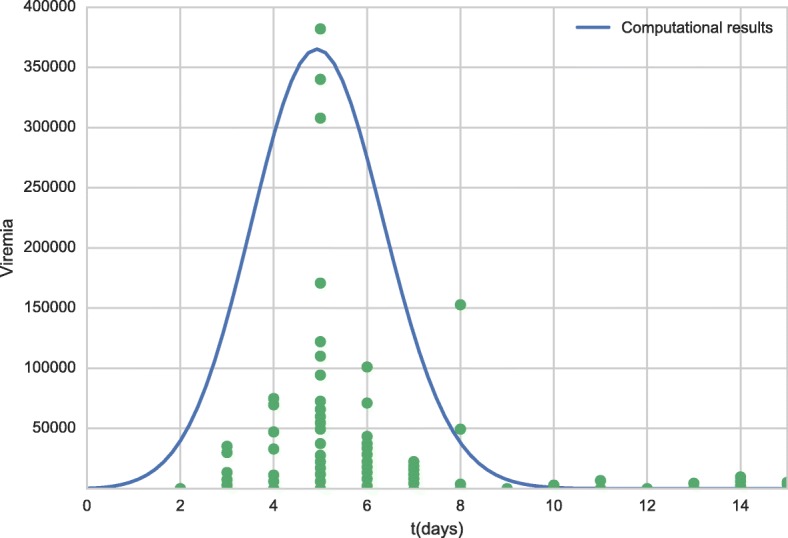
Viremia curve for all period obtained by model (line) and experimental data obtained from the literature [32] (dots). Each dot in time scale represents a distinct patient

Figure 3 shows the viremia curve obtained by the simulation of the model in comparison with the experimental data obtained from the literature [32].

### Booster dose

**Fig. 4:**
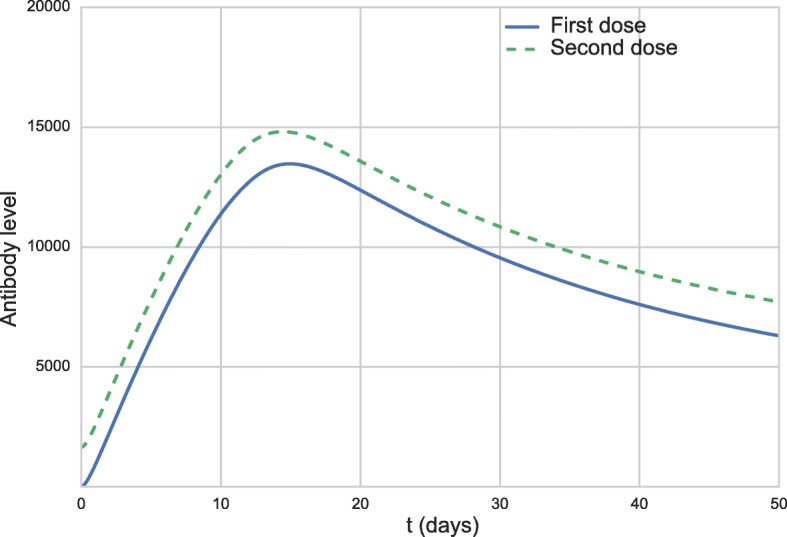
Antibody curves 50 days after the first vaccination (full blue line) and after the booster dose (dashed green line)

**Fig. 5:**
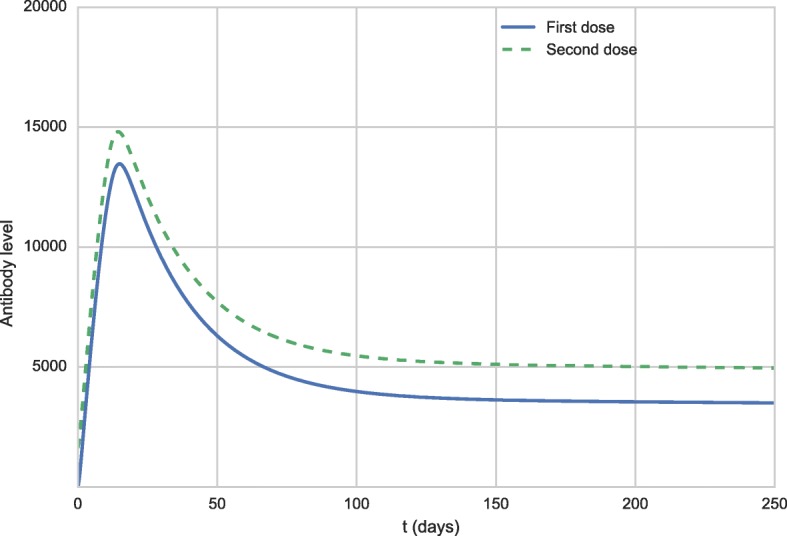
Antibody curves 250 days after the first vaccination (full blue line) and after the booster dose (dashed green line)

The administration of a booster dose was simulated 10 years after the administration of the first dose. The simulation is quite simple. As in the previous scenario, the initial value of *V* was set to 27,476 to simulate the administration of the first dose. Then, the simulation is executed until day number 3,650, when the value of variable *V* is set again to 27,476. The difference from the beginning of the simulation is that this time antibodies and memory cells that were produced after the first dose are present. Figures 4 and 5 present the antibody curves. The results of the booster dose simulation were shifted to facilitate its comparison to the results of the first dose. The solid line curve represents the response to the first dose, while the dashed curve represents the response to the booster dose.

**Fig. 6:**
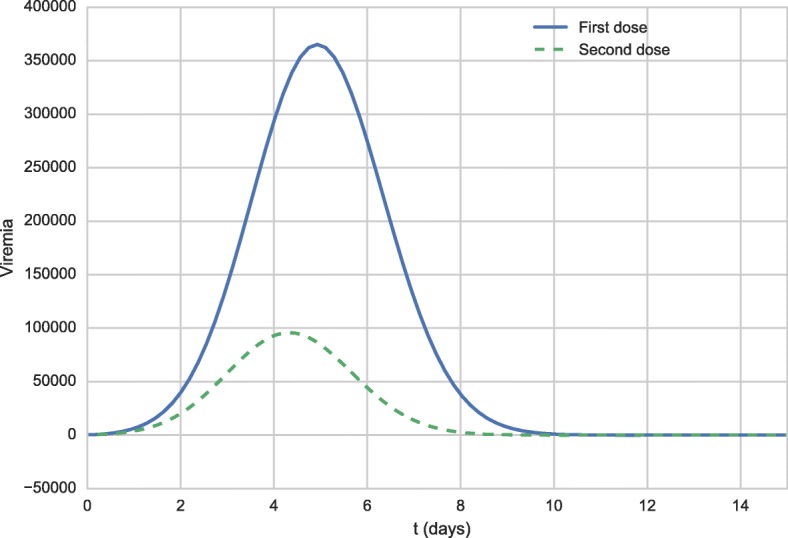
Viremia curves 15 days after the first vaccination (full blue line) and after the booster dose (dashed green line)

Figure 6 shows the viremia curves 15 days after the administration of the vaccine.

### Naïve T CD8+ levels

The clearing of the intracellular pathogen via CD8+ cytotoxic T lymphocytes appears to be important for recovering from primary viral infection. Based on this observation found in the literature [57], the authors decided to compare the immunological response given by the simulation of the model with different levels of CD8+ T cells in order to evaluate the impact of this population of lymphocytes on viral clearance.

**Fig. 7:**
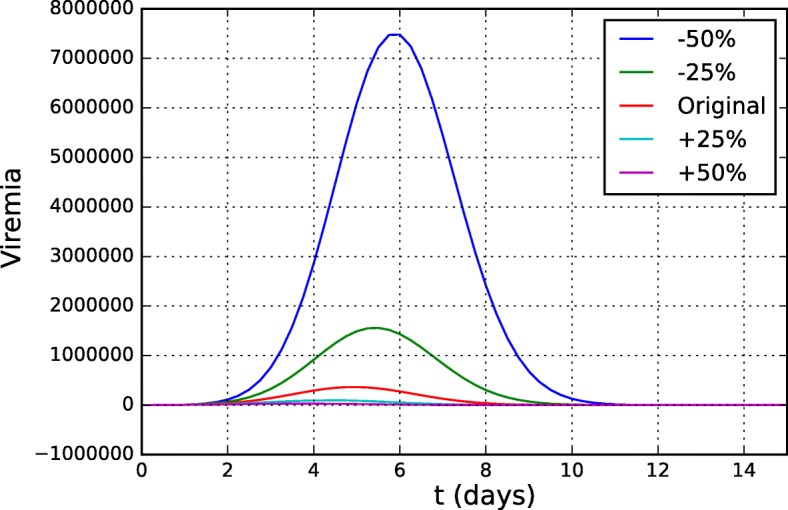
Viremia curves for different initial conditions of CD8+ T cells

**Fig. 8:**
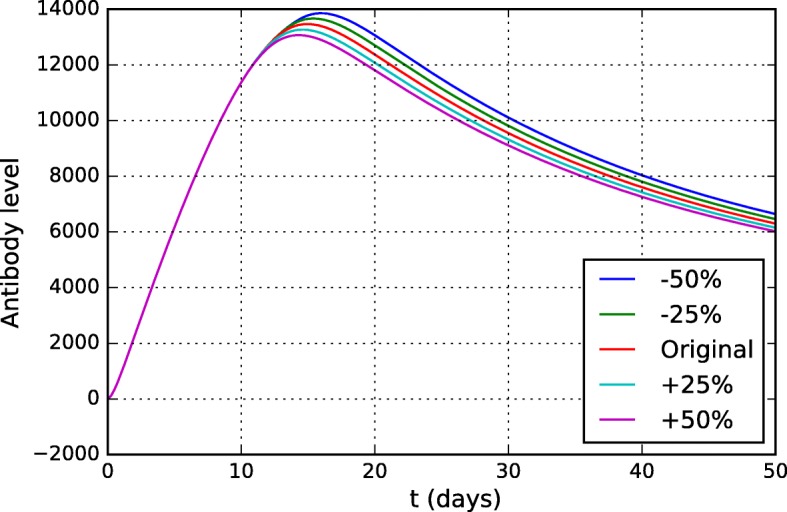
Curves of antibody levels obtained by the 50-day simulation of the model, for different initial values of CD8+ T cells

**Fig. 9:**
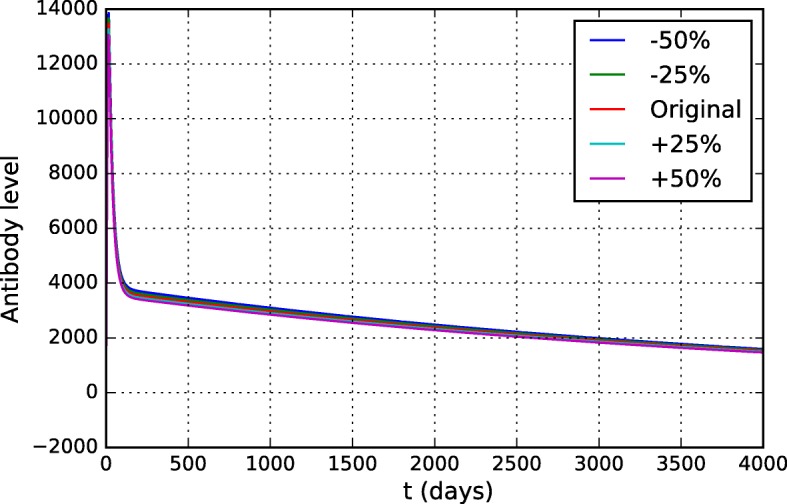
Curves of antibody levels obtained by the 4,000-day simulation of the model, for different initial conditions of CD8+ T cells

Figure 7 shows the viremia curves for different initial CD8+ T cell values, and Figs. 8 and 9 show antibody levels.

### Dose-response

An experimental work [32] reported that “doses from 27,476 IU to 587 IU induced similar seroconversion rates and neutralizing antibodies geometric mean titers (GMTs)”. Based on this study, a second scenario analyzes the results of our model when different dose values are administered. The values used in the simulation are the same to those used by the experimental work [32]: 31 IU, 158 IU, 587 IU, 3,013 IU, 20,447 IU and 27,476 IU.

**Fig. 10:**
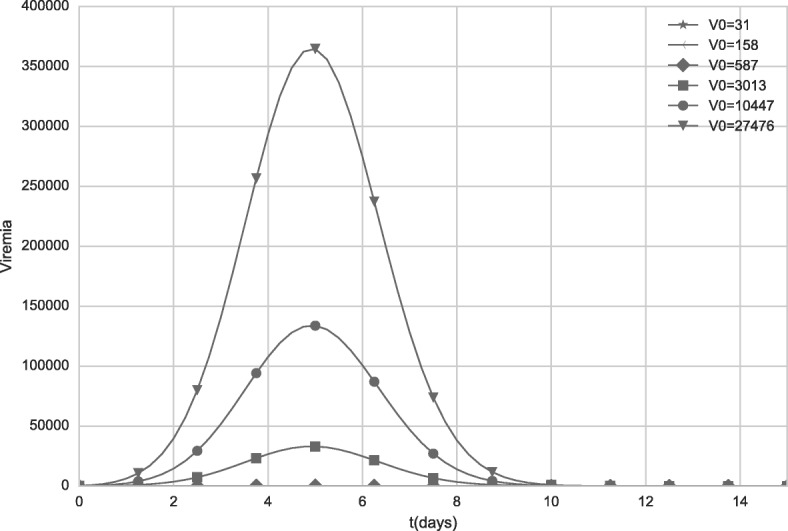
Viremia curves obtained by the model when distinct initial values of V (vaccine virus) are used. The values represent distinct vaccine doses. For doses equal to 31 IU and 158 IU, viremia was equal to zero

**Fig. 11:**
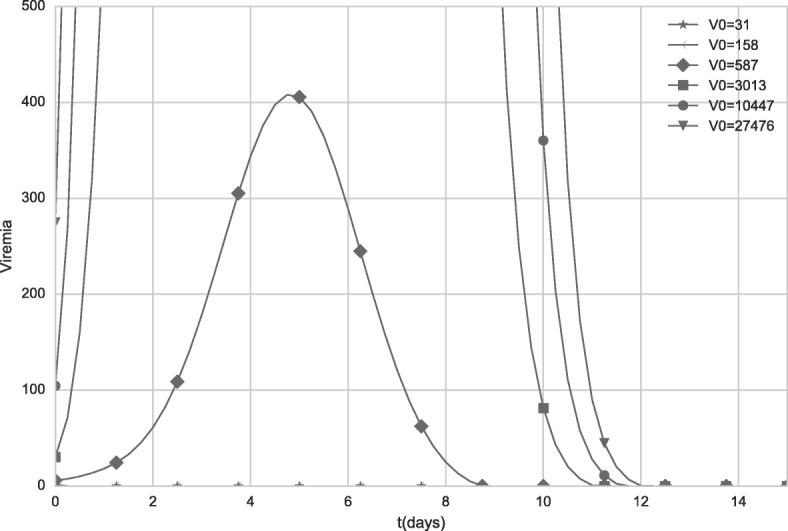
Viremia curves obtained by the model when distinct initial values of V (vaccine virus) are used. The values represent distinct vaccine doses. The scale was changed to better illustrate the viremia induced after administration of a dose with 587 IU. For doses equal to 31 IU and 158 IU, viremia was equal to zero

Figures 10 and 11 show the viremia curves obtained by the model for distinct vaccine doses. Figure 11 uses a smaller scale to allow the visualization of the simulated viremia curve obtained after administration of the dose with 587 IU (represented by diamonds).

**Fig. 12:**
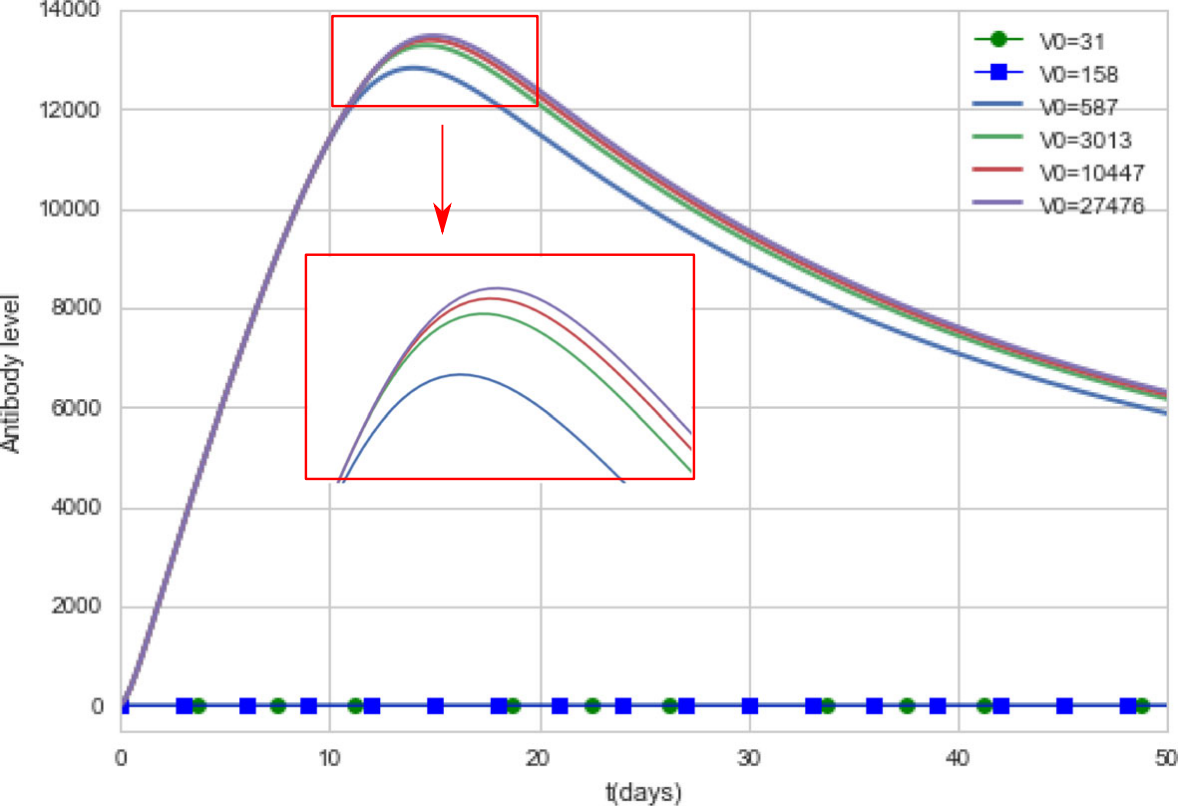
Antibody curves generated by the computational model. The model simulates the antibody concentrations during a 50-day period for different doses of the vaccine. For doses equal to 31 IU and 158 IU, the antibody curves were equal to zero

**Fig. 13:**
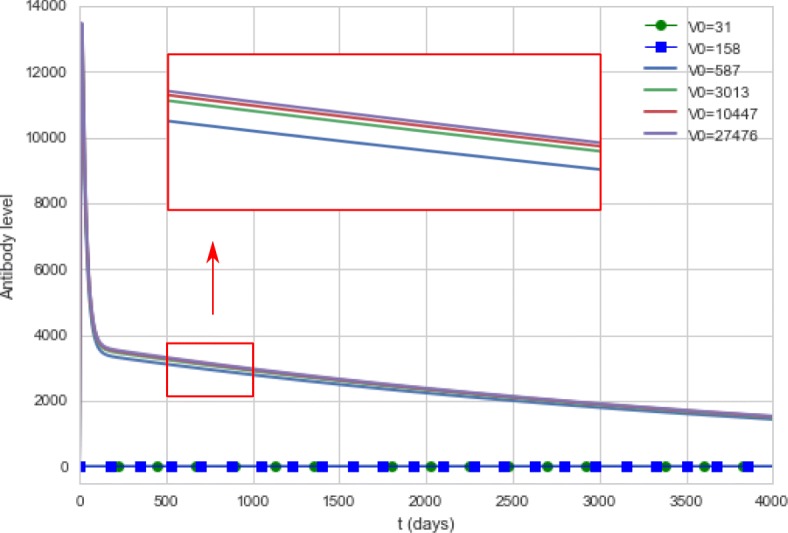
Antibody curves generated by the computational model. The model simulates the antibody concentrations during a 4,000-day period for different doses of the vaccine. For doses equal to 31 IU and 158 IU, the antibody curves were equal to zero

Figure 12 presents the antibody curves generated by the computational model during a 50-day period for different doses of the vaccine, while Fig. 13 presents the antibody curves obtained by simulating 4,000 days after vaccination.

Doses using 31 IU and 158 IU did not produce viremia nor antibody titers, so the curves are superimposed on the x-axis.

### Sensitivity analysis

The sensitivity analysis identifies the impact caused by the variation of parameters and initial conditions of the mathematical model in the dependent variables [59]. If a small change in a parameter is responsible for a drastic change in the result of the problem, it means that the problem is sensitive to that particular parameter. Otherwise, this parameter has a low impact on the model. This analysis is used to help the understanding of the mathematical model, since it allows the identification of the most relevant parameters, that is, the values of these parameters must be carefully defined.

A brute-force approach was used to examine the influence of all parameters of the model. The parameter values were varied from -10% to + 10% (in 5% intervals) from their original values. The original values are presented in Table 3 and were obtained after adjustment using experimental data from the literature [32, 58, 60, 61]. For each parameter, the curves that simulate the level of antibodies and viremia were evaluated, since they are the main populations of interest and on which there is experimental data. Only the parameters to which the model was most sensitive will be presented in this section.

**Fig. 14:**
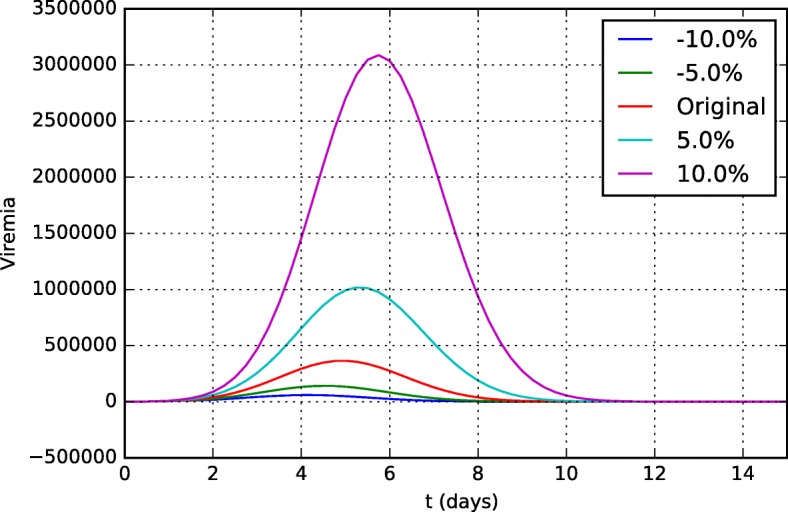
Sensitivity analysis of the parameter *π*_*v*_ in the viremia curves 15 days after vaccination

As expected, the model was sensitive to most of the parameters of the equation describing the virus dynamics (Eq. 1). The antibody curves were not significantly affected by them, therefore only the viremia curves will be presented. Figure 14 shows the distinct viremia curves obtained for different values of *π*_*v*_. This parameter represents the viral replication rate and, as one could expect, the model was very sensitive to it. The more the virus can multiply, the more difficult it is for the HIS to contain it and the higher the viremia level is.

**Fig. 15:**
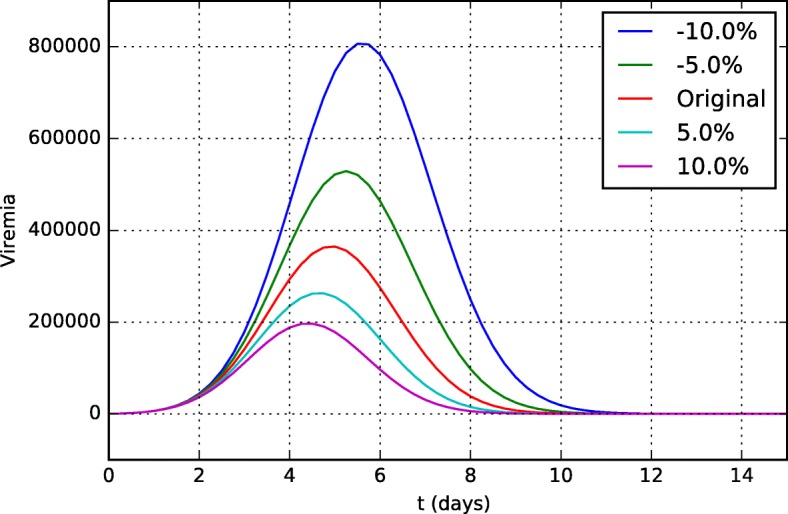
Sensitivity analysis of the parameter *k*_*v*1_ (neutralization rate of YF virus per unit of neutralizing antibodies) in viremia curves 15 days after vaccination

**Fig. 16:**
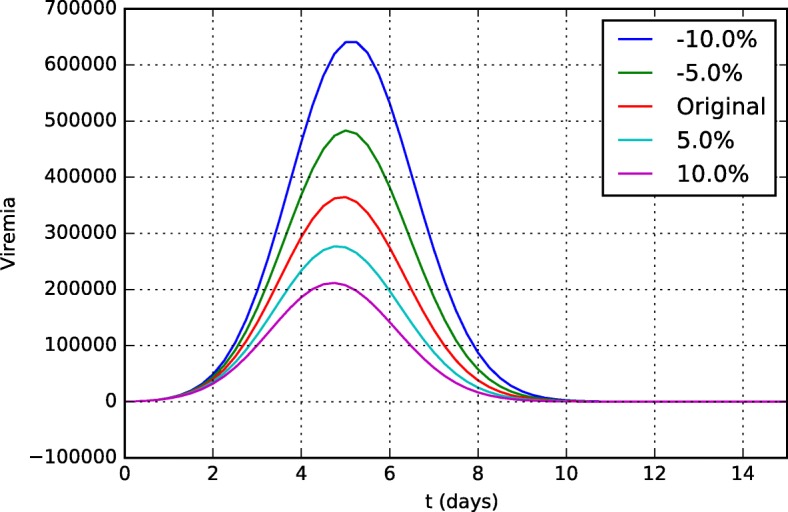
Sensitivity analysis of the parameter *k*_*v*2_ (neutralization rate of YF virus per unit of CD8+ T cell) in viremia curves 15 days after vaccination

Figures 15 and 16 present the viremia curves obtained by simulation of the model for different values of *k*_*v*1_ and *k*_*v*2_, respectively. These parameters represent the neutralization rates of the YF virus per unit of neutralizing antibodies (*k*_*v*1_) and CD8+ T cells (*k*_*v*2_).

If we consider that the parameter *k*_*v*1_ represents the ability of antibodies to neutralize the YF virus, its value can be understood as its affinity/specificity to the YF virus and, if it is more specific and can neutralize the virus better, viral replication will be better controlled and viremia will be lower.

It is easy to understand why the model, especially the viremia curve, is so sensitive to parameter *k*_*v*2_. It represents the ability of CD8+ T cells to induce apoptosis of an infected cell. Thus, the higher this ability, the fewer the number of infected cells. Since YF viruses use infected cells to reproduce themselves, the viremia level is reduced.

## Discussion

As can be observed from Figs. 1 and 2, from a qualitative point of view, the values obtained from the computational experiments are very close to the experimental results. Also, the literature reports that the antibody concentration in the bloodstream peaks at about two weeks after vaccination [62], a value close to the one obtained in the computational experiments.

Figure 3 shows that, in the simulation, the peak viremia value occurs on the fifth day, consistent with the literature, which reports that it occurs between four and six days after vaccination [63], as well as with experimental results [32]. In addition, the literature reports that ten days after vaccination, viremia is undetectable [63], which is consistent with the computational results. For some patients, however, viremia can be detectable, as one experimental result has shown [32].

Figures 4 and 5 show that the behavior described in the literature [57] resembles that obtained by the simulation of the model: the neutralizing antibodies levels are slightly increased after the booster dose.

The viremia curves shown in Fig. 6 demonstrate that viremia reaches much lower levels after the administration of the booster dose than those seen after the administration of the first dose. Although the viremia level is lower after the administration of the booster dose than the first dose, it is not possible to say that this level is below the threshold of detectable viremia as described in the literature: “viremia has not been documented in persons receiving a booster dose of YF vaccine” [64]. This occurs because of the use of distinct units to measure viremia, and the fact that it is not trivial to convert one unit to another. For this reason, this work considers only qualitative results, and not quantitative ones. This result still needs to be quantitatively compared to experimental data in order to better validate our model, but the qualitative behavior presented is satisfactory since the level of antibodies and/or memory cells was able to contain viral replication more efficiently than it was observed for a naïve individual. Furthermore, the viremia level was almost 4 times lower for the booster dose than for the first dose.

As show in Fig. 7, as the number of CD8+ T cells is reduced, the viremia increases and lasts longer, reinforcing the importance of CD8+ T cells in the control of viral replication. Figures 8 and 9 show that this variation in initial CD8+ T cell values did not significantly affect antibody production nor duration of immunity.

As observed in the Figs. 10 and 11, all doses greater than 3,013 IU produce high levels of viremia. Although the viremia increases with the use of doses with higher concentrations, the antibody response presents a very subtle difference, as can be observed in Fig. 12. The 587 IU dose, which presented a much smaller, unremarkable viremia (Fig. 10) compared to the doses with higher concentrations, was also able to induce an antibody response similar to that induced by formulations with higher concentrations.

Figure 13 suggests that the duration of immunity does not appear to be affected by vaccine formulations with distinct concentrations: all doses above 587 IU present similar results. For now, it seems that yellow fever vaccine can be used in much lower doses than usual: the computational experiments indicate that vaccine formulations with 587 IU can produce the same seroconversion rates than the 27,476 IU formulation, in accordance to the experimental data [32]. Although the reference paper investigated the duration of immunity for a smaller period of time, approximately 10 months after vaccination [32], its conclusions were similar to those obtained by the computational experiments: “GMTs of each group were not statistically different from the reference vaccine". The computational results are also in agreement with other studies. One paper [57] concluded: “there was no correlation between the level and duration of detectable 17D viremia and the postvaccination nAb level". Another paper presents a similar conclusion [65]: “the serological response was not related to virus dose as the titres obtained with high or low doses of virus was at the same level".

The results of the simulations for these four scenarios have shown that the model was able to reproduce, from a qualitative perspective, clinical results reported in the literature, despite all simplifications [50]. Some aspects not directly included in the model may be indirectly present, as constants. Therefore, the choice of distinct values for a constant may represent distinct behaviors in the system. For example, one paper [58] points out that “The decreasing trend in antibody titres with the time since vaccination appeared strongly modified by age". In our model, the effects of age in the production of antibodies could be reproduced increasing or decreasing the values used for the antibody secretion rate (Eq. 10, *π*_*AS*_ and *π*_*AL*_). For this reason, our model does not need to include age as one of its parts. The same applies for other aspects of the immune system that are not directly included in the set of equations.

In this work we consider that the vaccine does not cause adverse events, such as Yellow fever vaccine-associated viscerotropic disease (YEL-AVD) and Yellow fever vaccine-associated neurotropic disease (YEL-AND), due to their rarity.

This model was developed and adjusted based on the immune system response to the YF vaccine, but it should be noted that the concept presented in the mathematical model is generic enough to represent the action of other diseases or vaccines in the HIS. For this, changes in both initial conditions and parameters values are probably needed.

Obtaining experimental data to adjust and validate the model is not a trivial task. Studies on the duration of immunity are difficult to interpret because different groups use distinct methods to evaluate seroprotection. There is no well-established serological value of protection in humans and cellular immunity data are very scarce. Also, as stated above, the values reported for viremia use distinct units, which cannot be converted into other units due to the different methods used to obtain such data. These factors made it difficult to obtain experimental data compatible with the standards and units used in the model presented in this work, and consequently to use more studies available in the literature to adjust and validate it.

Although the results found are qualitatively in agreement with the few experimental data found in the literature, more tests and refinement of the model may be needed to adjust it. To do so, experimental data to better validate the simulated scenarios needs to be obtained, in particular for booster dose and CD8+ T cells. With this, it would be possible to also evaluate the model from a quantitative perspective and, if necessary, to better adjust it. With more data available, the model may be improved, making it more reliable and sufficiently accurate to be used to help answer open questions about YF vaccine.

One of the next steps in this work is to reintroduce CD4+ T lymphocytes in the model. This could be important to simulate the effects of the YF vaccine in immunosuppressed individuals, such as people living with HIV. Since many of the YF endemic countries are in Africa, where the HIV infection rates are also high, the investigation of the best YF vaccination scheme for these individuals is relevant, since they have, in general, fewer CD4+ T lymphocytes, which are important to the activation of other lymphocytes and consequently to the production of antibodies. This population deserves special attention because the YF vaccine is made with live virus, therefore the risk of systemic lethal infection exists.

## Conclusion

This work presented the qualitative validation of a reduced mathematical-computational model to represent the immune response to the YF vaccine using four distinct scenarios. The first one simulates the immune response to the administration of the standard dose of the 17DD-YFV. The second one simulates the immune response to distinct doses of vaccine. The third scenario simulates the administration of a booster dose ten years after the first dose. Finally, we evaluated the impact of changing the CD8+ T cells values. The results of a sensitivity analysis of the model was also shown. Two populations, virus and antibodies, were the main focus of the simulations because more experimental data are available and qualitative behaviors are described in the literature for these populations. The results of the simulations were collected and compared to the values reported in the literature. From a qualitative point of view, the results obtained by the computational model satisfactorily reproduced the clinical results.

## Abbreviations

ABM: Agent-based models
APCs: Antigen presenting cells
BDF: Backward differentiation formula
DRC: Democratic Republic of Congo
EYE: Eliminate yellow fever epidemics
GMT: Geometric mean titers
HIS: Human immune system
HIV: Human immunodeficiency virus
IDE: Integrated development environment
IU: International units
ODEs: Ordinary differential equations
YF: Yellow fever
YEL-AND: Yellow fever vaccine-associated neurotropic disease
YEL-AVD: Yellow fever vaccine-associated viscerotropic disease
